# Long‐term exposure to air pollution and repeated measures of cognitive function among cognitively normal adults – impact of physical activity

**DOI:** 10.1002/alz70860_103501

**Published:** 2025-12-23

**Authors:** Anke Huels, Lina Dimitrov, Tong Wen, Stefanie Ebelt, Donghai Liang, Allan I. Levey, James J. Lah, Thomas S. Wingo

**Affiliations:** ^1^ Emory University, Atlanta, GA, USA; ^2^ University of California, Davis, Sacramento, CA, USA

## Abstract

**Background:**

Despite the established association between fine particulate matter (PM_2.5_) exposure and cognitive decline, the critical time window of exposure and potential counteracting effects of physical activity are not well understood. We investigated associations between long‐term PM_2.5_ exposure up to 15 years prior to cognitive testing, physical activity, and cognitive function in a cognitively normal mid‐life population.

**Method:**

The ongoing Emory Health Brain Study recruits cognitively normal individuals >50 years. Participants undergo cognitive testing every two years with neuropsychological assessments of memory, executive function, and language. Lower z‐scores indicate worsening cognitive function. Residential long‐term exposure to PM_2.5_ elemental carbon, ammonium, sulfate, organic carbon, and nitrate 3, 5, 10 and 15 years prior to enrollment was estimated with a spatial resolution of 50m‐1km. We used commercial residential records from LexisNexis to account for residential history. Associations between PM_2.5_ components and repeated cognitive testing were estimated using quantile g‐computation adjusting for age, gender, race, ethnicity, education, area deprivation index, and time trend. Effect modification analyses were conducted for physical activity at baseline.

**Results:**

We analyzed 3,564 observations from 1,941 participants (up to 4 visits). Mean age at baseline was 63±7 years, 66% were female. Increasing all PM_2.5_ components in the 15‐year average exposure by one quartile was associated with lower cognitive test results for memory (e.g., ‐0.07 [95%‐confidence interval: ‐0.10, ‐0.04] lower delayed recall test) and executive function (‐0.06 [‐0.10; ‐0.02] lower number span backward test) but not for language (e.g., ‐0.02 [‐0.07; 0.02] lower multilingual naming test). Associations were strongest for longer exposure windows, e.g., ‐0.04 [‐0.08; 0.00] for the average 3‐year exposure and ‐0.06 [‐0.10; ‐0.02] for the average 15‐year exposure for executive function. According to their weights in the quantile g‐computation analysis, ammonium and sulfate were the most influential components. Associations were significantly attenuated (interaction *p*‐value=0.018) among those who exercised at least 3 days per week (0.00 [‐0.08; 0.09] for executive function) in comparison to those who did not (‐0.09 [‐0.14; ‐0.05]).

**Conclusion:**

Long‐term PM_2.5_ components exposure was associated with lower performance in memory and executive function. Being physically active was protective against the adverse effects of air pollution.

